# Intelligence

**Published:** 1828-10

**Authors:** 


### INTELLIGENCE.

### MONTHLY REPORT OF PREVALENT DISEASES.

The complaints met with in London during the last month have scarcely assumed such a decided character as to enable us to point out any as the *prevailing* disease. Those to which our attention has principally been directed have been cases of diarrhœa, occasionally, but not generally, accompanied with vomiting,—and continued fever, for the most part mild, but in some instances severe. Upon the whole, we think there is more disease in London at present than during the wet weather which so long prevailed in the earlier part of the season. There has been nothing to remark in the treatment of these fevers; and, with regard to the diarrhœa, we shall only observe, that there are very few cases which resist the Hydrargyrum cum Creta, either alone, or (when the stomach is not too irritable,) combined with Dover's powder. The usual dose has been five grains of each three times a day.

---

### *Royal College of Surgeons in London.*

*Collegial Anatomical Prize.*—The Board of Curators announce, that the subject for the third prize is "An Inquiry into the ultimate Terminations of the Sanguiferous System, and the Commencements and Terminations of the Lymphatic System; explanatory of the means by which parts of the body are formed, maintained, altered, and removed; authenticated, as far as practicable, by Preparations."

Candidates to be members of the College,—not of the Council.

Dissertations to be in English.

Each Dissertation to be distinguished by a motto or device; and accompanied by a sealed paper, containing the name and residence of the author, and having, on the outside, a motto or device corresponding with that on the Dissertation.

Dissertations to be addressed to the Secretary, and delivered at the College before Christmas day 1830.

The manuscript Prize Dissertation, and every accompanying drawing and preparation, will become the property of the College.

Dissertations which shall not be approved, with their accompanying drawings and preparations, and their correspondent sealed papers, will be returned, upon authenticated application, within the period of three years; and those manuscripts which shall remain three years unclaimed, together with their accompanying drawings and preparations, will become the property of the College; at which period the papers containing the names of the respective authors will be burned, unopened, in presence of the Board of Curators.

By order of the Board,

EDMUND BELFOUR, *Sec.*

*Lincoln's Inn Fields; 5th day of August,* 1828.

---

*Jacksonian Prize: R. Coll. of Surgeons, London.*—But one of the two prizes for the year 1827 having been adjudged, two prize-subjects are proposed for the year 1829, viz. "Encysted Tumors," and "Bronchocele."

Candidates to be members of the College.

Dissertations to be in English; and the number and importance of facts will be considered principal points of excellence.

Each Dissertation to be distinguished by a motto or device; and accompanied by a sealed paper, containing the name and address of the author, and having, on the outside, a motto or device corresponding with that on the Dissertation.

Dissertations to be addressed to the Secretary, and delivered at the College before Christmas day 1829.

The manuscript Prize Dissertation, and every accompanying drawing and preparation, will become the property of the College.

Compositions which shall not be approved, with their accompanying drawings and preparations, and correspondent sealed papers, will be returned, upon authenticated application, within the period of three years; and those manuscripts which shall remain three years unclaimed, and every accompanying drawing and preparation, will become the property of the College; at which period their accompanying papers, containing the names of the respective authors, will be burned, unopened, in the presence of the Jacksonian Committee.

The prize-subject for the present year, 1828, is "The Causes, Consequences, and Treatment of Inflammation of the several distinctions of Membrane." Dissertations upon which must be delivered at the College before Christmas day next.

By order,

EDMUND BELFOUR, *Sec.*

*Lincoln's Inn Fields; 19th day of August*, 1828.

---

*Regulations of the Council, respecting the Reading Room of the Royal College of Surgeons in London.*—The reading room of the College shall be open from ten till four every day, except on Saturdays and Sundays; except also during the month of August.

Members and articled students of the College shall be admitted to the reading room.

Other persons, desirous of admission, shall send their applications in writing, (specifying their christian and surnames, rank or profession, and places of abode,) to the Board of Curators.

Admission will be granted for six months, at the expiration of which time application shall be made for a renewal of the permission.

All possible despatch shall be used in supplying readers with such books as they may require.

Readers taking extracts from any book, shall not lay the paper on which they write on any part of such book: nor shall any tracings be taken from any plate without the permission of the Board of Curators.

Readers are not to write on any part of a book belonging to the College; and if any one should observe a defect in any book, he is requested to communicate the same to the librarian.

N.B. The admission tickets are by no means transferable; nor may readers introduce friends, or amanuenses, without respective tickets.

By order,

EDMUND BELFOUR, *Secretary.*

*27th December*, 1827.

### *Cambridge Examinations.*

In our last Number we gave insertion to a letter, signed "Justus," in answer to one bearing the signature of "Verax" in our Number for August. Verax has answered the letter alluded to in the pages of a contemporary and we subjoin his protest against the accuracy of Justus, on the old principle of stating both sides of the question.

*To the Editor of the London Medical Gazette.*

Sir,—The Medical and Physical Journal of the 1st September contains a letter, signed Justus, which comments on some observations of mine in the preceding Number accompanying the copies of the examination for the degree of M.B. at Cambridge.

I am well acquainted with the discipline of that university, and with the medical examinations during the last eight years, and also with the system pursued at Edinburgh and in London; and not ignorant of the nature of the medical examinations in the other capital cities of Europe. The charge, therefore, of ignorance of the subject falls to the ground.

I have now, therefore, to refer to the other charge—the only alternative left me by Justus—of being "uncandid."

I repeat, I am well acquainted with the university and its institutions: it is the first time I have ever heard "that many of the most difficult questions were not expected to be answered at all;" and, from my personal knowledge, I can aver that the assertion is not correct.

The examination is a *bonâ fide* examination, as much so as that in Edinburgh or in London; and the best proof of this is, that candidates are not unfrequently rejected from the very circumstance of not answering questions which Justus would make us believe were only *pro formâ:* I am acquainted with instances of such rejection.

In every examination, whether at Edinburgh or in London, a candidate is not rejected if he answers, either imperfectly or not at all, one or two out of many questions, unless such questions are so easy as to make ignorance of them quite disgraceful. In the same manner, at Cambridge, a man would not be rejected for not answering a difficult question as to a process in pharmacy, or an equivalent number in chemistry, although his ready answer to such questions would give a very favorable idea of the way in which he had employed his time, in addition to his knowledge on other subjects more immediately applying to medical science.

Justus observes, "a printed list of all the questions are given him on his admission, and, by subsequent reference or study, he may become capable of understanding the whole." Not one word of this do I comprehend, for not one word of it is correct. The candidate has no knowledge whatever of the questions to be put to him previous to his entering the examination room, except that he is to have questions in anatomy, physiology, practice of physic, pharmacy, and chemistry, with portions of Hippocrates and Celsus for translation. It is needless to observe, that, neither in the schools nor in the professor's rooms, where these examinations are conducted, are there any means of reference whatever.

There is an insinuation, if possible, still more incorrect than these gross mis-statements; viz. "that these questions are selected, that, by their subsequent publication, the importance of a university education may be inferred from the difficult examination which the candidates are hastily supposed to have passed."

The simple fact that these examinations have been instituted at least eight years; that the questions have, until lately, not even been printed, and never till last year I believe published, until I sent them to the Medical and Physical Journal, is the best answer to such an observation. I need hardly observe, that, were there the slightest foundation for such an observation, it would stamp the character of Professor HAVILAND with obloquy, (a name above reproach,) and taint even that of the university.

I have then, sir, only to repeat, that the examination is a *bonâ fide* examination; that the questions are expected to be answered; and that, if the majority are not answered,—aye, and well too,—the candidate is not admitted to his degree.

I retract, therefore, sir, not one word contained in my first letter; and, if it be necessary, I can bring forward men of the highest character and honour, members of the university, who, like myself, have personal knowledge of these facts, who are ready to give their testimony to that statement being essentially correct. I am, sir, your obedient servant,

VERAX.

---

*New Effervescing Chalybeate.*—Messrs. LAMING and Co., of Bishopsgate street, have contrived a saline aperient, (very much resembling Cheltenham salts,) which is administered in a state of effervescence, and which we are informed makes an excellent substitute for the common Seidlitz powder.

---

*Return, shewing the Number of Pupils at different Anatomical Schools.*

| | 1826. | | | | 1827. | | | |
|---|---|---|---|---|---|---|---|---|
| | No. of Pupils entered to Anatomy. | No. to Dissecting Room. | No. who have actually Dissected. | No. of Bodies actually Dissected. | No. of Pupils entered to Anatomy. | No. to Dissecting Room. | No. who have actually Dissected. | No. of Bodies actually Dissected. |
| St. Bartholomew's Hospital, Mr. Abernethy | 172 | 150 | — | 65 | 176 | 160 | — | 85 |
| Guy's Hospital, •• Mr. B. Cooper | 84 | 86 | 151 | 80 | 81 | 88 | 142 | 86 |
| St. Thomas's Hospital, Mr. Green | 167 | — | 121 | 70 | 133 | — | 145 | 70 |
| London Hospital, Mr. Headington | 60 | 45 | 40 | 42 | — | — | — | — |
| Great Windmill-street, Mr. Mayo | 79 | 71 | 95 | 90 | 82 | 60 | 94 | 70 |
| Webb-street School, Borough, Mr. Grainger | 125 | 125 | — | 112 | 142 | 142 | — | 95 |
| Little Dean-street, Soho, Mr. Bennett | 38 | 30 | 30 | 30 | | | | |
| Chapel-street, Grosvenor-square, Mr. Sleigh | 50 | 50 | 45 | 15 | | | | |
| Dean-street, Soho, .. Mr. Carpue | 30 | 30 | 20 | | | | | |
| Howland-street, Fitzroy-square, | | | | — | | | | |
| Mr. Tuson | 17 | 17 | 14 | 8 | | | | |
| Aldersgate-street, •••• Mr. Tyrrell | 42 | 46 | 46 | 28 | 46 | 55 | 54 | 39 |
| Little Windmill-st. Mr. Dermott | 45 | 51 | 30-35 | 30-35 | 50 | 43 | 30-35 | 30-35 |

Mr. Lawrence's *Election into the Council.*—Mr. Lawrence has been elected a member of the council of the College of Surgeons. The nomination is said to have been made by Mr. Abernethy, and seconded by Sir A. Cooper. It is also said that the council were much divided on the subject of his admission; but the general opinion seems to be that his election is calculated to advance the interests of the profession, by removing some of those subjects of dissention which have of late years disturbed it.

---

### ANATOMY.

We subjoin the Report of the Committee appointed by Parliament to inquire into the sources of the difficulties in procuring the requisite supply of subjects for dissection. The document is drawn up with great care, and is the result of much and laborious investigation. It does not admit of curtailment without injury, and we have therefore presented our readers with it at full length. The principal suggestions thrown out are—that the law should be repealed which consigns the bodies of murderers to dissection; and that those dying in institutions supported by the public should, under certain restrictions, be used for anatomical purposes. By these means it is supposed the prejudices against the practice would gradually be got rid of, and the disgusting expedient of exhumation entirely abolished. Some change in the present state of matters is imperatively called for; as it appears, besides the great difficulty of getting the requisite supply of subjects, that there is scarcely a student or teacher of anatomy throughout the kingdom who, under the present law, "is not indictable for a misdemeanour."

The number of witnesses examined was very great, and comprehended persons of various ranks and professions; and, although their evidence differs in some particulars, there is nevertheless an astonishing accordance on the most important points.

*Report of the Select Committee on Anatomy.*

The Select Committee appointed to inquire into the manner of obtaining subjects for dissection in the schools of Anatomy, and into the state of the law affecting the persons employed in obtaining or dissecting bodies; and to whom several petitions for the removal of impediments to the cultivation of the science of anatomy were referred; and who were empowered to report the minutes of evidence taken before them; have, pursuant to the order of the House, examined the matters to them referred, and agreed to the following Report:

The peculiar nature of the subject which the committee were appointed to investigate, has induced them to inquire principally into the practice of the anatomical schools of London, where, by personal communication with the most eminent surgeons, and with the students and principal teachers of anatomy, it could be fully ascertained that no detriment to their interests was to be apprehended from the publicity to arise out of the present inquiry. With regard to the practice of the provincial schools, to avoid the expence of summoning witnesses from a distance, they have been satisfied with written communications from resident professors or practitioners of eminence, which will be found in the Appendix.

The committee have inquired into the nature of the difficulties which the anatomists have here to contend with, whether arising out of the state of the law, or an adverse feeling on the part of the people; and into the evil consequences thence ensuing, as well to the sciences of medicine and surgery, as to all who study, teach, and practise them, and eventually to the members of the whole community. They have called witnesses to shew in what manner the wants of the anatomist are provided for in several foreign schools, and to state their opinion whether similar methods could be applied with advantage in this country, and, if applied, would be adequate to remove the present difficulties.

The first origin of these difficulties is obviously to be traced to that natural feeling which leads men to treat with reverence the remains of the dead; and the same feeling has prompted them, in almost all times and countries, to regard with repugnance and to persecute anatomy.

As the importance of the science to the well-being of mankind was discovered, the governments of different states became its protectors, and in this country particularly, by the statute of Henry the VIIIth, protection to a certain extent was given, and intended to be given, to it; but that protection, which at first perhaps was fully adequate, owing to the rapid progress of the science, has long since become wholly insufficient.

How limited were the wants of the science in the former part of the last century, may be learnt from the lectures of Dr. William Hunter, who describes the professors of the most celebrated schools, both at home and abroad, as employing in each course of lectures not more than one, or at most two, subjects, and as exhibiting the performance of the operations of surgery, not on human bodies, but on those of animals. He represents the students in medicine and surgery as never exercising themselves in the practice of dissection, because for such practice they had no opportunities.

For such a system of instruction the provisions of the statute of Henry the VIIIth might well be adequate, and these provisions, indeed, may now be considered of importance only as a distinct admission of the principle that the government of this country ought to protect anatomy. The reformation of this antiquated and imperfect system took place, in this country, in the year 1746, when Dr. William Hunter, having a singular enthusiasm for the science, established complete courses of anatomical lectures, and opened a regular school for dissection. The reform thus introduced was complete, and its author exulted before his death in having raised and diffused such a spirit for dissection, that he should leave behind him many better anatomists than himself.

Under his immediate pupils, and their successors, this school has gone on increasing. The earliest account that the committee have met with of the number of anatomical students resorting to London, is that given by Mr. Abernethy, who states that, shortly after the breaking out of the war with France, they amounted to 200. One of the witnesses, Dr. Macartney, computes their number, in the year 1798, at 300; and Mr. Brookes, a teacher of anatomy, in a calculation submitted to Sir Astley Cooper in the year 1823, then reckoned their number to be 1000. It appears from the returns now furnished by the teachers of the different schools in London, that the number at present is somewhat above 800; the diminution in the number since the year 1823 being the consequence, probably, of the pupils resorting to foreign schools, the advantages of which were less known at the former period than they are at present.

When it is considered what a demand there is for practitioners, as well to meet the wants of an increased population at home, as of an extended empire of colonies and dependencies abroad, this rapid increase of students will not appear surprising; and if it is considered, also, that not only is that demand an increasing one, but that every practitioner, however humble, from that laudable desire for intellectual improvement which characterises the present age, endeavours, if he can afford it, to obtain a good education, and must regard himself as ill educated if he has not gone through a course of dissection, the eventual increase of dissecting students can hardly be calculated, should their wants be supplied abundantly and at a cheap rate.

Although the students now attending the schools of anatomy in London exceed 800, not more than 500 of this number actually dissect. The duration of their studies in London is usually sixteen months, and during that time the number of subjects with which every student in surgery ought to be supplied, appears from the evidence (although there is some difference on this point) to be not less than three; two being required for learning the structure of the parts of the body, and one the mode of operating. The total number of subjects actually dissected in the schools of London, in one year, is stated to be not greater than from 450 to 500, which is after the rate of less than one subject for each dissecting student; a proportion wholly insufficient for the purposes of complete education.

Dissection, on an extended scale, began in this country before there existed any such general feeling in its favour, founded on an opinion of its utility, that the British government, after the example of some foreign governments, would venture openly to patronise it. Accordingly, when, in 1763, Dr. Hunter proposed to build an anatomical theatre, and to endow it with his museum and a salary for a professor, provided the government would grant him a site of ground for the institution, and his late Majesty would extend to it his countenance and protection, he met with a silent refusal. It was therefore only by stealth, and by means not recognised by the law, that the teacher was enabled to procure subjects. These means, it is notorious, from the time of Dr. Hunter down to the present time, have been principally disinterment; though, of late, other illegal modes and contrivances, such as stealing before burial, personation of relatives for the purpose of claiming bodies, &c. have occasionally been had recourse to. For some time after the first establishment of dissecting schools, while the number of teachers and students was small, and the demand for subjects very limited, the means which were resorted to for obtaining a supply were adequate to the wants of the students, and bodies were obtained in abundance and cheaply. The exhumators at that time were few, and circumspect in their proceedings; detection was rare; the offence was little noticed by the public, and was scarcely regarded as penal; so that (according to one of the witnesses) long after the decision of the judges, in 1788, that disinterment was a misdemeanour, prosecutions for this offence were not common, and offenders taken in the fact were usually liberated. If this state of things had continued, though the illegality of the practices had recourse to must be conceded, yet they could scarcely be said to occasion evils of such magnitude as to require a legislative remedy. But the number of students and teachers having greatly increased, and, with them, the demand for subjects and the number of exhumators, detections became frequent, the practice of exhumation notorious, and public odium and vigilance were directed strongly against the offenders. It may be collected from the debates in Parliament which took place in the year 1796, during the progress of a Bill for subjecting to dissection the bodies of felons executed for burglary and robbery, that even at that time the public regarded disinterment with strong feelings of jealousy.

In proportion as the public became vigilant, the laws relating to sepulture were interpreted and executed with increasing rigor; and, as the price of subjects rose with the difficulty of obtaining them, the premium for breaking the laws increased with the penalty. The exhumators increased in number, and, being now treated as criminals, became of a more desperate and degraded character.

The parties of daring men who now took to raising bodies, did it happen (as was frequently the case) that, while in pursuit of the same spoil, they fell in one with another, actuated by vindictive feeling, and regardless of the caution and secrecy on which the successful continuance of their hazardous occupation must depend, had contests in the places of sepulture,—left the graves open to public gaze,—or gave information to magistrates, or the relatives of the disinterred, against their rivals. Frequently, with a view to raise the price of subjects, to extort money, or to destroy rivalry, they have proceeded to acts of outrageous violence, tending to excite the populace against the teachers of anatomy. These, and similar acts of violence or imprudence, have been constantly bringing exhumation to light, and have exasperated the public both against the exhumator and the anatomist: and this to such a degree, that of late, in many cases, individuals, out of solicitude to guard the dead, have taken upon themselves to dispense with the laws of their country, and have fired upon parties attempting disinterment. Other circumstances, but of minor importance, have been assigned by some of the witnesses as augmenting the difficulty of obtaining subjects in London, or increasing the demand for them; but, as regards them, the committee beg leave to refer to the evidence itself. The general result has been, with some difference, according to differences of place and season, (sometimes owing to the caprice and mercenary motives of the agents employed, at other times owing to the real difficulty of obtaining a supply,) that, of late, subjects have been to be procured, either not at all or in very insufficient quantity, and at prices most oppressive to the teacher and student.

The price of a subject, about thirty years ago, was from one to two guineas; the teacher now pays from eight to ten guineas; and the price has risen even to sixteen guineas. The teachers deliver subjects to their dissecting pupils at a lower price than that at which they purchase them, having been compelled to resort to this expedient, lest dissection in London should be abandoned altogether. The loss which they thus sustain is made good out of the fees which they receive for attendance on their lectures in the anatomical theatre. The cost of providing subjects is also enhanced to the teacher, by his being required occasionally to defend the exhumator against legal prosecution, and to maintain him against want, if sentenced to imprisonment, and his family, (in case he has one,) until the period of his punishment expires.

Nor is it only of a precarious, insufficient, and expensive mode of obtaining subjects, that the cultivators of anatomy complain: it is by the law, not as regards the exhumators, but as it affects themselves, that they are aggrieved.

The first reported case of a trial for disinterment is that of Rex *v.* Lynn, in the year 1788, when the Court of King's Bench, on a motion for an arrest of judgment, decided it to be a misdemeanour to carry away a dead body from a churchyard, although for the purpose of dissection, as being an offence *contra bonos mores* and common decency. In this state the law on the subject of disinterment, as interpreted by the Court of King's Bench, appears to have remained until the present year; when Davis and another were tried and convicted at the assizes at Lancaster, and subsequently received the sentence of the Court sitting at Westminster, for having taken into their possession, with intent to dissect, a dead body, at the time knowing the same to have been unlawfully disinterred. A respectable teacher of anatomy, residing at Liverpool, had been tried and found guilty on a similar indictment at the quarter sessions at Kirkdale, in the month of February in the same year. With these exceptions, magistrates appear hitherto to have taken no cognizance of receiving into possession a dead body, unless there were strict evidence that the receiver was a party to the disinterment; and on this practical view of the state of the law professional men also appear hitherto to have acted. At present, however, a most intelligent magistrate, one of the witnesses, considers that very slight evidence would connect the receiver with the disinterment; and that the purchase from the exhumator would suffice to send the case to a jury, the knowledge of the fact of disinterment being to be collected from the circumstances, if strong enough to justify the inference. It is stated that there is scarcely a student or teacher of anatomy in England who, under the law, if truly thus interpreted, is not indictable for a misdemeanour.

According to the opinion of the last-cited witness, to be a party to the non-interment, as well as to the disinterment of a dead body, would render a person indictable for a misdemeanour. Two cases are cited in support of this opinion. In the one, Rex *v.* Young, a non-reported case, but referred to by the Court in the case of Rex *v.* Lynn, the master of a workhouse, a surgeon, and another person, were indicted for, and convicted of, a conspiracy to prevent the burial of a person who died in the workhouse. In the other, Rex *v.* Cundick, which occurred at the Surrey spring assizes, in the year 1822, the defendant was found guilty on an indictment for a misdemeanour, charging him with not having buried the body of an executed felon, entrusted to him by the gaoler of the county for that purpose, but with having sold the body for lucre and gain, and for the purpose of being dissected: and, on this trial, it was not considered necessary to prove that the body had been sold for lucre, or for the purpose of dissection. The witness infers, from the analogy of all these cases, that to treat a dead body as liable to any thing but funeral rites, is an offence *contra bonos mores*, and therefore a misdemeanour.

This state of the law is injurious to students, teachers, and practitioners, in every department of medical and surgical science, and appears to the committee to be highly prejudicial to the public interests also.

It is the duty of the student to obtain, before entering into practice, the most perfect knowledge he is able of his profession, and for that purpose to study thoroughly the structure and functions of the human body; in which study he can only succeed by frequent and repeated dissection. But his wants cannot be adequately supplied in this country, except at an expence amounting nearly to a prohibition, which can be afforded only by the most wealthy, and precludes many students from dissecting altogether. From the precariousness or insufficiency of the supply, the dissections and lectures are often suspended for many weeks, during which the pupils are exposed to the danger of acquiring habits of dissipation and indolence; and, from the same causes, that important part of surgical education is usually omitted, which consists in teaching how to perform on the dead body those operations which the student may afterwards be required to practise on the living. Not only does the student find dissection expensive and difficult of attainment; but he cannot practise it, without either committing an infringement of the law himself, or taking advantage of one committed by others. In the former case, he must expose himself to imminent hazard; and, in either, he may incur severe penalties, and be exposed to public obloquy. The law, through the medium of the authorities entrusted with conferring diplomas, and of the Boards deputed by them to examine candidates for public service, requires satisfactory proof of proficiency in anatomical science, although there are no means of acquiring that proficiency without committing daily offences against the law. The illegality and the difficulties attending the acquisition of the science dispose the examiners, in some cases, to relax the strictness of their examination, and induce them, in the case of the Apothecaries' Company, to dispense with dissection altogether: the persons to whom certificates are granted by the examiners of this company being those who, from their numbers* and extensive practice, ought especially, for the safety of the public, to be well instructed. The annual number of certificates so granted exceeds 400.

The teacher of anatomy, besides the evils which befal him in common with the student, has to suffer others, arising also out of the state of the law, which affect him with peculiar hardship. The obstacles which impede the study of anatomy in this country are such, and the facilities presented to the study in foreign countries are so great, that those English students who are desirous of obtaining a thorough knowledge of the science desert the schools at home, and repair to those abroad. Their principal resort is to Paris, where 200 English students of anatomy are now pursuing their course of instruction. Dissection, probably, under these circumstances, would scarcely be followed at home, were it not for the regulations of the College of Surgeons, which require the candidates for the diploma of the College to have learned the practice of surgery in a recognised school within the United Kingdom; so that the student, during the period required for learning this practice, in order that he may the sooner become qualified for his profession, employs a part of his time in learning also to dissect. These disadvantages, affecting the teacher, are such, that, except in the most frequented schools, attached to the greater hospitals, few have been able to continue teaching with profit, and some private teachers have been compelled to give up their schools. To the evils enumerated it may be added, that it is distressing to men of good education and character to be compelled to resort, for their means of teaching, to a constant infraction of the laws of their country, and to be made dependent for their professional existence on the mercenary caprices of the most abandoned class in the community.

But it is not only to the student, while learning the rudiments of the science, and to the teacher, while endeavouring to improve it, that dissection is necessary, and the operation of the law injurious. It is essential also to the practitioner, that, during the whole course of his professional career, he should dissect, in order to keep up his stock of knowledge, and to practise frequently on the dead subject, lest, by venturing to do so unskilfully on the

* Computed at 10,000 in England and Wales.

living, he expose his patients to imminent peril. He is required also, in many important cases, civil and criminal, to guide the judgment of judges and of jurors, and would be rebuked were he to confess, upon any such occasion, that, from having neglected the practice of dissection, he was unable to throw light upon a point at issue in that science which he professed. He is liable, in a civil action, to damages for errors in practice, due to professional ignorance; though at the same time he may be visited with penalties as a criminal for endeavouring to take the only means of obtaining professional knowledge.

Under these circumstances, affecting equally the student, teacher, and practitioner, the committee were not surprised to find that this inquiry excited considerable interest in all parts of the country, and that numerous petitions from all classes of the profession, connected with the science of anatomy, were laid upon the table of the House, uniformly praying for an amendment of the existing law on the subject.

But, independently of the bearings of the question on the interests of medical practitioners, and on the health of the community, the system pursued is productive of great evil, by training up a race of men in habits eminently calculated to debase them, and to prepare them for the commission of violent and daring offences. The number of persons who, in London, regularly live by raising dead bodies, is stated, by the two police officers examined before the committee, not to exceed ten; but the number of persons occasionally employed in the same occupation is stated by the same witnesses to be nearly 200. Nearly the whole of these individuals, as is admitted by the exhumators themselves who were examined before the committee, are occupied also in thieving, and form the most desperate and abandoned class of the community. If, with a view to favor anatomy, exhumation should be allowed to continue, it appears almost a necessary consequence that thieves also should be tolerated. It should seem useless, however, with a view to suppress exhumation, to endeavour to execute the existing laws with increased severity, or to enact new and more rigorous ones. The effect of interpreting and executing the laws with increasing rigor has been, not to suppress exhumation, but to raise the price of bodies, and to increase the number of exhumators. So long as there is no legalised mode of supplying the dissecting schools, so long the practice of disinterment will continue: but, if other measures were devised, which would legalise and ensure a regular, plentiful, and cheap supply, the practice of disinterring bodies, and of receiving them, would, of necessity, be entirely abandoned.

Before adverting to those new methods for obtaining an adequate supply of subjects, which have been suggested by the witnesses who have been examined before the committee, they will state in what manner, according to the evidence adduced, the schools of anatomy at Paris are provided. They have also inquired into the practice of some other foreign schools, for an account of which they beg to refer to the evidence itself; and they dwell upon the practice of the schools of Paris, because it approaches most nearly to the plan recommended by most of the witnesses for adoption in this country.

The administration of all the hospitals at Paris, since the period of the revolution, has been confided to a public board of management. The rule at the hospitals is, that every patient who dies shall be attended by a priest, and that, after the performance of the usual ceremonies of the Catholic church, the body shall be removed from the chapel attached to the hospital, to the dead room, and there remain for twenty-four hours, if not sooner claimed by the relatives. Bodies may be examined after death, by the medical officers attached to a hospital, in order to ascertain the cause of death; but may not be dissected by them. A body, if claimed by the friends after examination, is sewed up in a clean cloth, before being delivered to them. If not claimed within twenty-four hours after death, after being enveloped in a cloth in a similar manner, it is sent, in the manner hereafter described, to one of the dissecting schools.

There are no private dissecting schools at Paris, but two public ones; that of the Ecole de la Medicine, and that adjoining the Hôpital de la Pitié. These are supplied exclusively from the different hospitals and from the institutions for maintaining paupers, the supply from certain of these establishments being appropriated to one school, and that from the remaining establishments to the other.

The distribution of subjects to the two schools is confided to a public officer, the Chêf des travaux Anatomiques. He causes them to be conveyed from the hospitals, at an early hour, in a covered carriage, so constructed as not to attract notice, to a building at the schools, set apart for that purpose. They are then distributed by the prosecteurs to the students; and, after dissection, being again enveloped in cloth, are conveyed to the nearest place of interment.

The students at the Ecole de la Medicine consist of young men who have distinguished themselves at a public examination, though the person at the head of the establishment is also allowed to admit pupils to dissect. The school of la Pitié is open to students of all nations, who, on entering themselves, may be supplied with as many subjects as they require, at a price varying, according to the state in which the body is, from three to twelve francs; priority of choice, however, being given to the *eleves internes* of the different hospitals, and the subjects being delivered to them at a reduced price. English surgeons were here permitted, until lately, to engage private rooms for the purpose of lecturing on anatomy to students of their own nation, and to superintend their labours in the dissecting room. From the protection and facilities which have thus been afforded to the study of anatomy at Paris, it has become the resort of the medical students of all nations; the practice of exhumation is wholly unknown, and the feelings of the people appear not to be violated.

It is the opinion of almost all the witnesses that the adoption in this country of a plan similar in most respects to that which prevails in France, would afford a simple and adequate remedy for the existing evils. They recommend that the bodies of those who during life have been maintained at the public charge, and who die in workhouses, hospitals, and other charitable institutions, should, if not claimed by next of kin within a certain time after death, be given up, under proper regulations, to the anatomist: and some of the witnesses would extend the same rule to the unclaimed bodies of those who die in prisons, penitentiaries, and other places of confinement. In the hospitals which supply subjects to the anatomical schools of France and Italy, religious rites are paid to the dead, before giving up the bodies for dissection: in the plan proposed for this country, most of the witnesses recommend that the performance of religious rites should be deferred until after dissection, and they are anxious that the anatomist should be required, under adequate securities, or a system of effective superintendence, to cause to be administered, at his own expense, to the bodies which he dissects, religious solemnities and the usual rites of burial.

The plan proposed has this essential circumstance to recommend it, that, provided it were carried into effect, it would yield a supply of subjects that, in London at least, would be adequate to the wants of the anatomist. The number of anatomical students resorting annually to London, and the number of subjects with which they ought to be supplied, have been already stated. It appears from the returns obtained by the committee, from 127 of the parishes situate in London, Westminster, and Southwark, or their immediate vicinity, that out of 3,744 persons who died in the workhouses of these parishes in the year 1827, 3,103 were buried at the parish expense; and that, of these, about 1,108 were not attended to their graves by any relations. There are many parishes in and around London from which, at the time of making this report, returns had not been delivered in; but it may be inferred from those returns which have been procured, that the supply to be obtained from this source alone would be many times greater than that now obtained by disinterment; that, when added to the supply to be derived from those other sources which have been pointed out, it would be more than commensurate to the wants of the student, and consequently that the plan, if adopted, as meeting the exigencies of the case, would eventually be the means of suppressing the practice of exhumation.

If it be an object deeply interesting to the feelings of the community that the remains of friends and relations should rest undisturbed, that object can only be effected by giving up for dissection a certain portion of the whole, in order to preserve the remainder from disturbance. Exhumation is condemned as seizing its objects indiscriminately,—as, in consequence, exciting apprehensions in the minds of the whole community, and as outraging in the highest degree, when discovered, the feelings of relations. If selection, then, be necessary, what bodies ought to be selected but the bodies of those who have either no known relations whose feelings would be outraged, or such only as, by not claiming the body, would evince indifference on the subject of dissection. It may be argued, perhaps, that the principle of selection, according to the plan proposed, is not just, as it would not affect equally all classes of the public; since the bodies to be chosen would necessarily be those of the poor only. To this it may be replied, first, that even were the force of this objection to a certain degree admitted, yet that, to judge fairly of the plan, its inconveniences must be compared with those of the existing system; which system, according to the evidence adduced, is liable in a great measure to the same objection, since the bodies exhumated are principally those of the poor; secondly, that the evils of this, or of any other plan to be proposed on this subject, must be judged of by the distress which it would occasion to the feelings of surviving relations; and the unfairness to one or another class of the community, by the degree of distress inflicted on one class rather than another; but, where there are no relations to suffer distress, there can be no inequality of suffering, and consequently no unfairness shown to one class more than another.

One or two of the witnesses, who appear to be either favorable or not opposed to the principle of the plan, speak with doubt of its success, as though it would be found impracticable to reconcile the public to its introduction; and one, in particular, apprehends that religious feelings may impede its the feelings of the person who w^d. [illegible] their burial [illegible]

adoption. An objection founded on religious feelings does not apply to the plan in question only, but would be equally valid, generally, against all dissection whatsoever; and should lead those who urge it, consistently with their own principles, to endeavour to put down altogether the study of practical anatomy.

Though it may be true that the public are, to a certain degree, averse to dissection, yet it is satisfactory to find several of the witnesses adducing facts to prove that those feelings of aversion are on the decline. They state that in those parish infirmaries where the bodies of those who die are examined, as the practice has become common, it has been viewed with less jealousy; that in those hospitals where a similar rule prevails, neither patients themselves are deterred from applying for admission, nor their relatives on their behalf; that the addition of public dissecting rooms to hospitals has not produced any diminution in the number of applications for relief within the walls of those hospitals; and that, by reasoning with the friends of those who die, and by explaining to them how important it is to the art of healing that examination should take place after death, they may usually be brought to consent to the bodies of their friends being examined. Hence it is argued that, in involving the subject of dissection in mystery, as has hitherto been the case, the public have been treated injudiciously; that, with proper precautions, and the light of public discussion to guide them, they may be made to perceive the importance of the study generally, and the reasonableness of the particular measure now contemplated, and that, when they come to regard it as the means of suppressing exhumation, they will receive it with favor, and finally acquiesce in it.

The legislative measure which most of the witnesses are desirous of, in order to enable them to carry the plan into effect, is the repeal of any existing law which would subject to penalties those who might be concerned in carrying the proposed plan into execution: they wish for an enactment, permissive and not mandatory, declaring that it shall not be deemed illegal for the governors of workhouses, &c. and for anatomists, the former to dispose of, the latter to receive and to dissect, the bodies of those dying in such workhouses, &c.; such bodies not having been claimed, within a time to be specified, by any immediate relations, and due provision being made for the invariable performance of funeral rites. Some few of the witnesses, indeed, who state that they wish for the success of the plan, contemplate any legislative interference whatever in this matter with apprehension; but they do not appear to have been aware how nearly the cases decided by the courts of law, and already adverted to, would apply to persons engaged in executing the plan in question. In those cases the bodies, for the non-burying of which the defendants were severally convicted, were those of a pauper who died in a workhouse, and of a person who had suffered death as a felon. If these cases apply, as it appears they do, to persons engaged in giving up or in receiving, for other purposes than for burial, the bodies of the inmates of workhouses or of prisons, such impediments to the success of the plan cannot be removed, as these witnesses think they might be, simply by the favorable interference of the executive government, however disposed to show indulgence to the profession; but an Act of the legislature can alone provide a remedy.

Amongst the measures that have been suggested for lessening the dislike of the public to dissection, is that of repealing the clause of the Act of George

II. which directs that the bodies of murderers shall be given up to be anatomised. It appears from the returns already laid before the House, that, as regards the direct operation of this clause on the supply of subjects, the number which it yields to the anatomist is so small in comparison of his total wants, that the inconvenience which he would sustain from its repeal would be wholly unimportant. As to its remote operation, almost the whole of the witnesses examined before the committee, and of those whose written communications will be found in the Appendix, are of opinion that the clause in question, by attaching to dissection the mark of ignominy, increases the dislike of the public to anatomy, and they therefore are desirous that the clause should be repealed.

The committee would be very unwilling to interfere with any penal enactment which might have, or seem to have, a tendency to prevent the commission of atrocious crimes; but, as it may reasonably be doubted whether the dread of dissection can be reckoned amongst the obstacles to the perpetration of such crimes, and as it is manifest that the clause in question must create a strong and mischievous prejudice against the practice of anatomy, the committee think themselves justified in concluding that more evil than good results from its continuance.

The committee consider that they would imperfectly discharge their duties if they did not state their conviction of the importance to the public interests of the subject of their inquiries. As the members of the profession are well educated, so is their ability increased to remove or alleviate human suffering. As the science of anatomy has improved, many operations formerly thought necessary have been altogether dispensed with; most of those retained have been rendered more simple; and many new ones have been performed, to the saving of the lives of patients, which were formerly thought impossible. To neglect the practice of dissection, would lead to the greatest aggravation of human misery; since anatomy, if not learned by that practice, must be learned by mangling the living. Though all classes are deeply interested in affording protection to the study of anatomy, yet the poor and middle classes are the most so: they will be the most benefited by promoting it, and the principal sufferers by discouraging it. The rich, when they require professional assistance, can afford to employ those who have acquired the reputation of practising successfully. It is on the poor that the inexperienced commence their practice, and it is to the poor that the practice of the lower order of practitioners is confined. It is, therefore, for the interest of the poor especially that professional education should be rendered cheap and of easy attainment; that the lowest order of practitioners (which is the most numerous), and the students on their first entry into practice, may be found well instructed in the duties of their profession.

Such, on an attentive consideration of the evidence adduced, is the deliberate judgment of the committee on the matters submitted to them; and it now remains for the House to consider whether it will not be expedient to introduce, in the course of the ensuing session, some legislative measure, which may give effect to the recommendations contained in the present Report.

*22d July*, 1828.

### METEOROLOGICAL JOURNAL,

*From August 20th, to September 20th, 1828.*

By Messrs. HARRIS and Co. Mathematical Instrument Makers, 50, High Holborn.

| August | Rain gauge. | Moon. | Thermom. | | | Barometer. | | De Luc's Hygrom. | | Winds. | | Atmospheric Variations. | | |
|---|---|---|---|---|---|---|---|---|---|---|---|---|---|---|
| | | | 9 A.M. | MAX. | MIN. | 9 A.M. | 10 P.M. | 9 A.M. | 2 P.M. | 9 A.M. | 10 P.M. | *9 a.m.* | *2 p.m.* | *10 p.m.* |
| 20 | | | 66 | 70 | 60 | 30.00 | 29.88 | 49 | 49 | WNW | W | Fine | Fine | Fine |
| 21 | .03 | | 66 | 67 | 58 | 29.76 | .66 | 49 | 49 | W | NW | Fine | Rain | — |
| 22 | .07 | | 64 | 63 | 54 | .62 | .70 | 49 | 49 | WNW | N | Fine | Rain | Rain |
| 23 | | | 61 | 62 | 51 | .88 | .96 | 49 | 49 | NW | N | Fine | Fine | Fine |
| 24 | | | 61 | 66 | 63 | 30.07 | 30.08 | 49 | 49 | NW | NW | — | — | — |
| 25 | | ○ | 69 | 72 | 71 | .14 | .18 | 50 | 51 | NW | NE | — | — | — |
| 26 | — | | 74 | 73 | 55 | .21 | .20 | 51 | 51 | E | SE | — | — | — |
| 27 | | | 72 | 71 | 59 | .18 | .14 | 50 | 50 | SE | SE | — | — | — |
| 28 | | | 72 | 73 | 59 | .11 | .08 | 50 | 50 | SE | SE | — | — | — |
| 29 | | | 69 | 72 | 60 | .07 | .05 | 50 | 50 | SE | SE | — | — | — |
| 30 | | | 65 | 70 | 57 | .06 | .04 | 49 | 50 | E | NE | — | — | — |
| 31 | | | 66 | 69 | 56 | .01 | 29.89 | 49 | 50 | ESE | ESE | Cloudy | Cloudy | Cloudy |
| Sept. | | | | | | | | | | | | | | |
| 1 | | ☾ | 65 | 69 | 55 | 29.84 | .84 | 50 | 50 | ESE | ESE | Rain | Cloudy | Fine |
| 2 | | | 66 | 67 | 56 | .91 | .92 | 50 | 50 | SE | NE | Fine | Fine | Fine |
| 3 | .08 | | 64 | 66 | 58 | .94 | .94 | 50 | 50 | E | E | Rain | Rain | Fine |
| 4 | | | 64 | 67 | 58 | .93 | .93 | 50 | 50 | E | SE | Fine | Fine | Fine |
| 5 | | | 66 | 68 | 58 | .91 | .91 | 51 | 51 | SE | SE | Fine | Cloudy | Fine |
| 6 | | | 64 | 67 | 58 | .96 | .90 | 51 | 51 | SE | SE | Fine | Fine | Fine |
| 7 | | | 63 | 65 | 54 | .90 | .81 | 50 | 51 | SE | SSE | — | — | — |
| 8 | | | 66 | 74 | 53 | .83 | .70 | 50 | 49 | S | SE | — | — | — |
| 9 | .02 | ● | 65 | 74 | 54 | .74 | .79 | 49 | 51 | SW | WSW | Rain | Fine | — |
| 10 | .24 | | 64 | 68 | 58 | .65 | .45 | 52 | 50 | SSE | WSW | Fine | Rain | Cloudy |
| 11 | .16 | | 67 | 64 | 61 | .45 | .47 | 50 | 51 | W | W | Cloudy | Show'ry | Fine |
| 12 | .46 | | 62 | 65 | 58 | .40 | .33 | 52 | 55 | WSW | W | Rain | Cloudy | Rain |
| 13 | .09 | | 64 | 68 | 52 | .46 | .57 | 51 | 51 | NW | NW | Fine | Fine | Show'ry |
| 14 | .22 | | 54 | 58 | 50 | .70 | .90 | 51 | 50 | NW | NE | Rain | Show'ry | Cloudy |
| 15 | | | 53 | 60 | 46 | 30.17 | 30.32 | 50 | 49 | E | ENE | Fine | Fine | Fine |
| 16 | | ☽ | 53 | 59 | 42 | .40 | .38 | 48 | 47 | NE | SE | — | — | Cloudy |
| 17 | | | 54 | 62 | 53 | .37 | .08 | 47 | 48 | SE | SE | — | Cloudy | Cloudy |
| 18 | | | 57 | 64 | 45 | 29.93 | 29.91 | 48 | 48 | SE | SE | — | Fine | Fine |
| 19 | | | 50 | 67 | 48 | .96 | 30.03 | 48 | 49 | ESE | SE | — | — | — |

The quantity of Rain fallen in the month of August, was 2 inches and 80.100ths.

